# Health Services Delivery by Non-physicians and Associated Factors in Iran: A Cross-sectional Study in 2023

**DOI:** 10.34172/aim.31947

**Published:** 2025-04-01

**Authors:** Ahmad Mehri, Seyed Saeed Hashemi Nazari, Farideh Mostafavi, Mehran Rostami Varnousfaderani, Seyed Amirhosein Mahdavi, Reza Haj-Manouchehri, Seyed Davood Mirotorabi, Mohsen Saberi Isfeedvajani

**Affiliations:** ^1^Department of Epidemiology, School of Public Health and Safety, Shahid Beheshti University of Medical Sciences, Tehran, Iran; ^2^Legal Medicine Research Center, Legal Medicine Organization, Tehran, Iran; ^3^Spiritual Health Research Center, Life Style Institute & Department of Community Medicine, Faculty of Medicine, Baqiyatallah University of Medical Sciences, Tehran, Iran

**Keywords:** Healthcare services, Non-physician providers, Patient satisfaction, Traditional medicine

## Abstract

**Background:**

The increasing demand for healthcare services and some associated factors including lack of physicians, lack of trust in physicians, ineffectiveness of treatments and high costs may be have led to a rise in non-physician-provided services. This study aims to assess health services delivery by non-physicians and its associated factors in Iran.

**Methods:**

This study was a cross-sectional study conducted using a convenience sampling method in the Iranian community in 2023. A standard questionnaire with 45 questions was developed based on focus group discussions and a validation process to assess the status of receiving services in six medical areas including traditional medicine, abortion, traditional dentistry, obesity and slimming diets, bone setting, and addiction treatment. Data collection was carried out using online questionnaires on Iranian and non-Iranian social media platforms. Descriptive and analytical statistics were used to analyze the data, with logistic regression adjusting for various demographic factors.

**Results:**

Out of 1713 participants, 53.9% (95% CI: 51.5%-56.3%) were women, and the majority were in the 30-40 age group. Traditional Islamic medicine was the most commonly sought service, with 56% (95% CI: 51.2%-60.8%) of users receiving it from non-physicians. Satisfaction with non-physician services varied, with 32.1% (95% CI: 25.8%-38.4%) reporting high satisfaction for traditional medicine, but only 49.4% (95% CI: 40.5%-58.3%) for experimental dentistry. Key reasons for choosing non-physician providers included the effectiveness of traditional treatments and fear of modern medicine’s side effects.

**Conclusion:**

The result showed that the use of non-physician services can be considerable and that necessary interventions should be designed to standardize treatments and deal with substandard providers who may be harmful to the health of the community.

## Introduction

 Nowadays, health systems deal with increasing demand for healthcare services, shortage of healthcare workers, overload of tasks, overcrowding of health centers, care delays, long waiting lists, expensive healthcare costs and lack of regular access to affordable essential medicines and services, leading to seeking healthcare services from non-physician providers.^1^

 Traditional medicine is one of these services which are provided by both physicians and non-physicians, and it has increased in popularity worldwide over recent years.^2^ According to the World Health Organization, 75% of the world’s population uses herbal products for health purposes. Information on modern drug interactions is usually available in experimental studies with predefined regularity.^3^ However, interactions related to traditional medicines mostly remain untested and their efficacy is unconfirmed. In addition, non-physician providers are often uneducated about the potential toxicities of these medicinal plants.^4^

 Another service that is increasingly provided by non-physicians is beauty and dermatologic services. The dramatic increase in the demand for beauty services and the blur between medical procedures and beauty treatments have compelled non-physicians, in addition to dermatologists and plastic surgeons, to provide these services.^5^ Many procedures performed by non-physicians are unscientific and take place outside of the medical settings in a non-medical/health environment.^6^

 Therefore, the provision of these services in other environments and the provision of the procedures that take place there may cause concerns regarding compliance with the standards and regulations established to ensure the safety and protection of patients.^7^

 In Iran, like other developing medical services, some medical services are provided by non-physicians. In a study that was conducted in Tehran with the aim of comparing the amount of outpatient and inpatient use among physicians, informed consumers of health care services and non-physicians, uninformed consumers of health care services, the participants were randomly selected. The results of this study showed that in 349 physicians and 295 non-physicians, the amount of adjusted outpatient and inpatient use was higher in non-physicians (incidence ratio: 1.38 and 3.19, respectively; *P *value < 0.050). The only variable that had a significant relationship with the amount of consumption was the presence of chronic diseases because the incidence of hospitalization in patients with chronic diseases was 2.01 times that of people without diseases.^8^

 A study in Kashan, investigating the attitude of medical staff towards complementary medicine and medicine, showed that 60.9% of the participants were women. The average age of the study subjects was 29.70 ± 9.28 years. In total, 88.4% of the participants had no previous education in the field of complementary and traditional medicine, and 77.8% showed interest in learning in this field. Also, 57.6% of the participants had experience of personal use of complementary and traditional treatments.^9^

 Despite some limited studies, the status of receiving health services from non-physicians in Iran, as well as the factors associated with the use of non-physician services, remain unclear. Therefore, this study aimed to assess non-physicians’ health services and the reasons for using these services, in six medical areas including traditional medicine, abortion, traditional dentistry, obesity and slimming diets, bone setting, and addiction treatment.

## Materials and Methods

 This study was conducted in two stages using a mixed method. In the first stage, focus group discussions and expert opinions were used to prepare the questionnaire. First, based on a literature review, the reasons for requesting services by non-physician providers were extracted; then, a focus group interview was conducted with five experts (epidemiologist, medical specialist, forensic specialist, public health specialist, and medical ethicist). The interviews were conducted online in the environment of Google Meet. The discussions continued until the opinions were saturated. When all the research questions were discussed, the opinions were summarized and feedback from the participants was incorporated by presenting a summary of the points. The content analysis method was used to identify the patterns (themes) in the data. First, all the phrases and terms mentioned by the participants were transcribed completely word for word and were repeatedly reviewed and coded. Open coding was used to identify concepts, categories, or themes, and axial coding was used to connect between the concepts. Then, by selective coding, the themes were developed. Finally, the questionnaire was categorized and designed based on six structural concepts, including traditional medicine, abortion, traditional dentistry, obesity and slimming diets, traditional bone setting, and addiction treatment. The final questionnaire consisted of 45 questions that were viewable in an online form based on the type of service received by the participants. At the beginning of this questionnaire, the demographic characteristics of the individuals were collected. Finally, the participants were asked about receiving the mentioned services in the past year, and if the answer was yes, questions related to that service were asked, including the place of receiving the service, satisfaction with the service, the place of receiving the service, and the reason for referring to a non-physician. After preparing the initial version, the questionnaire was given to seven experts to determine its validity and reliability (one epidemiologist, one psychologist, one forensic medicine specialist, one health education specialist, and three laypeople) and was reviewed for appropriateness, clarity, comprehensiveness, simplicity, and fluency. The content validity ratio (CVR) and content validity index (CVI) indices for all questions in the questionnaire were above 0.7, and the validity of the questionnaire was confirmed. For internal reliability, the questionnaire was given to 30 people, and the Cronbach’s alpha index was calculated for each question and all questions. This index was also above 0.73 for all questions, which confirmed the reliability of the questionnaire.

 In the second stage, the link of the standard questionnaire was shared in different social media to complete the questionnaire via online survey in a cross-sectional study with an estimated sample size of 1530. The sample size was determined using the following formula:


$$\begin{array}{l} n=\frac{{\left(Z_{1-\alpha /2}\right)}^{2}p\left(1-p\right)}{d^{2}}\\ n=\frac{{\left(Z_{1-\alpha /2}\right)}^{2}0.2\left(1-0.2\right)}{0.02^{2}}=1530 \end{array}$$

With p = 0.20, type α error = 5%, and d = 0.02, the desired sample size was estimated to be 1,530 participants. Using the online questionnaire, 1,730 people participated in this study. The questionnaire included two parts. The first section included demographic characteristics for all participants. The second part included the questions related to the receipt of six services during the last year. The type of service received, the person performing the service, the reason for referring to a non-physician, the source of the non-physician referral, the location of the service receiving, the occurrence of complications after receiving the service, and the level of satisfaction with the service were asked from each of the participants.

 The sampling frame includes all active users of the target social media platforms (such as Instagram, Telegram, or other relevant platforms) in Iran in 2023, who have access to, experience with, or information about healthcare services provided by non-physicians. Study subjects were selected by convenience sampling. Data collection was carried out using online questionnaires on Iranian and non-Iranian social media platforms. First, the most popular channels and pages on Instagram, Telegram, EITA, and BALE were identified on these platforms. Then, an online questionnaire was published on these pages. First, a text was placed in the mentioned channels, which included the purpose of the study, the project’s code of ethics, and a written consent form. People were invited to enter this research by clicking on the questionnaire link. The questionnaire with all the questions related to all the mentioned services was posted on the social media platforms. For people who received any of the services during the past year, the specific questions related to that service were opened for them and they could answer the questions of that section.

 After collecting data through an online questionnaire, descriptive analysis was performed using number and percentage indicators. In the analytical part, the relationship between each of the demographic characteristics and the service provider was adjusted to other variables through multiple logistic regression. So, for the analysis, in the dependent variable, the basic level of referring to physician and the first level of referring to a non-physician were considered. First, the univariable liner regression analysis of each of the demographic variables with the outcome was analyzed. The variables that had a *P* value less than 0.2 were included in the multivariable linear regression analysis to determine the adjusted relationship to other variables. All analyses were performed at the 95% confidence level using STATA version 17.

## Results

 According to the results presented in Table 1, approximately 1,713 individuals participated in this study, of whom 927 (53.9%) were women and the remaining 46.1% were men. In terms of age, about one-third of the participants were in the 30-40 age group (29.9%), followed by the 20-30 age group, which had the second highest frequency (27.1%).

**Table 1:** Demographic Characteristics of Study Participants.

**Variable**	**Categories**	**Men** **789 (46.1)**	**Women** **927 (53.9)**	**Total** **1713**	***P *** **Value**
		**No. (%)**	**No. (%)**	**No. (%)**	
Age	< 20	39 (4.9)	17 (1.8)	56 (3.3)	< 0.001
	20-30	196 (24.8)	269 (29.2)	465 (27.1)	
	30-40	239 (30.3)	273 (29.5)	512 (29.9)	
	40-50	154 (19.5)	236 (25.5)	390 (22.8)	
	> 50	161 (20.5)	129 (14)	290 (16.9)	
Marital status	Single	220 (27.9)	342 (37)	562 (32.8)	< 0.001
	Married	569 (72.1)	582 (63)	1151 (67.2)	
Self-expressed socioeconomic status	Very bad	54 (6.8)	60 (6.5)	114 (6.7)	< 0.001
	Bad	226 (28.6)	234 (25.3)	460 (26.9)	
	Moderate	364 (46.1)	519 (56.2)	883 (51.5)	
	Good	132 (16.7)	104 (11.3)	236 (13.8)	
	Very good	13 (1.6)	7 (0.8)	20 (1.2)	
Self-reported health status	Very bad	10 (1.3)	7 (0.8)	17 (1)	0.232
	Bad	38 (4.8)	61 (6.6)	99 (5.8)	
	Moderate	320 (40.6)	400 (43.3)	720 (42)	
	Good	363 (46)	395 (42.7)	758 (44.2)	
	Very good	58 (7.4)	61 (6.6)	119 (6.9)	
Level of education	Middle school	22 (2.8)	20 (2.2)	42 (2.5)	0.021
	High school	32 (4.1)	12 (1.3)	44 (2.6)	
	Diploma/seminary education (preliminary course), diploma/pre-university	91 (11.5)	98 (10.6)	189 (11)	
	Associate's degree/Postgraduate diploma/seminary education (level 1 religious sciences)	39 (4.9)	47 (5.1)	46 (5)	
	Bachelor's/Bachelor's/seminary education (second level of religious sciences)	304 (38.5)	383 (41.5)	86 (40.1)	
	Master's Degree/Postgraduate Degree/Professional Physician/Seminary education (Third Level of Religious Sciences)	218 (27.6)	269 (29.1)	687 (28.4)	
	Specialized Physicianate /Post Physicianate /Seminary studies (first semester abroad)//Seminary studies (second semester)	83 ()	95 (10.3)	487 (10.4)	
Job	Retired	86 (10.9)	43 (4.7)	129 (7.5)	< 0.001
	Private sector	217 (27.5)	92 (10)	309 (18)	
	Government sector	271 (34.3)	451 (48.8)	722 (42.1)	
	Military department	19 (2.4)	7 (0.8)	26 (1.5)	
	Unemployed	42 (5.3)	46 (5)	88 (5.1)	
	Housewife	5 (0.6)	164 (17.7)	169 (9.9)	
	Soldier	22 (2.8)	-	22 (1.3)	
	Student	127 (16.1)	121 (13.1)	248 (14.5)	
Daily activity hours on social networks	< 1	101 (12.8)	129 (14)	230 (13.4)	0.060
	1-2 hours	290 (36.8)	308 (33.3)	598 (34.9)	
	2-3 hours	194 (24.6)	226 (24.5)	420 (24.5)	
	3-4 hours	117 (14.8)	119 (12.9)	236 (13.8)	
	> 4 hours	87 (11)	142 (15.4)	229 (13.4)	

 The majority of individuals were married (67.2%), and 51.5% reported having a moderate socioeconomic status, while only 15% reported having a good or very good socioeconomic status. Most participants reported their health status as good (44.2%), and 6.8% reported their health status as bad or very bad. In terms of educational level, bachelor’s degree/license/seminary education (second level of religious sciences) had the highest frequency (40.1%), and in terms of occupation, employment in the public sector (42.1%) and student (14.5%) had the highest relative frequency. Regarding daily activity hours on social networks, the 1-2 hour range (34.9%) and the 2-3 hour range (24.5%) had the highest frequencies. The difference observed in the distribution of people in all demographic characteristics, except for self-reported health and daily activity hours, was statistically significant between men and women.

 According to Table 2, out of a total of 406 people who received traditional Islamic medicine services in the past year, only 44% received services from physicians, while 52.5% received services from non-physicians. Of those who received services from non-physicians, 32.1% reported high or very high satisfaction, 48.4% reported moderate satisfaction, and 19.5% reported low or very low satisfaction.

**Table 2:** Status of Referral to a Non-physician/physician for Services Received During the Last Year

**Total Participants 1713**						
**Row**	**Service**	**Number of Recipients (Percentage of the Total)**	**Server Level**	**Number (percentage of Service Recipients)**	**Level of Satisfaction With non-physicians**	**Number (Percentage of Service Recipients)**
1	Traditional-Islamic medicine	406(23.7)	Physician	179 (44)	Very high/high	61 (32.1)
			Non-physician	213 (52.5)	Moderate	92 (48.4)
			Uncertain	14 (3.5)	Very low/low	37 (19.5)
2	Bone setting	64(3.7)	Physician	0 (0)	Very high/high	37 (57.8)
			Non-physician	64 (100)	Moderate	17 (26.6)
			Uncertain	0 (0)	Very low/low	10 (15.6)
3	Experimental dentistry	121(7.1)	Physician	0 (0)	Very high/high	60 (49.6)
			Non-physician	121 (0)	Moderate	40 (33.1)
			Uncertain	0 (0)	Very low/low	21 (17.3)
4	Abortion	29(1.7)	Physician	13 (44.8)	Very high/high	6 (37.5)
			Non-physician	16 (55.2)	Moderate	5 (31.2)
			Uncertain	0 (0)	Very low/low	5 (31.3)
5	Addiction Treatment	31(1.8)	Physician	6 (18.8)	Very high/high	9 (37.5)
			Non-physician	25 (81.2)	Moderate	7 (28)
			Uncertain	0 (0)	Very low/low	6 (24.5)
6	Obesity Treatment	310(18.1)	Physician	198 (63.9)	Very high/high	40 (35.7)
			Non-physician	112 (36.1)	Moderate	34 (30.4)
			Uncertain	0 (0)	Very low/low	38 (33.9)
7	Total number:	961(56.1)	-	-	-	-

 Among the 1713 participants, 64 (3.7%) used bone setting treatment services, with 57.8% of non-physician service users reporting high or very high satisfaction, 26.6% moderate, and 15.6% low or very low satisfaction. For experimental dental services, 121 participants (7.1%) used these services, with 49.6% reporting satisfaction, 33.1% moderate satisfaction, and 17.3% low or very low satisfaction.

 Out of 1713 participants, 29 (1.7%) received abortion services, with 37.5% reporting high or very high satisfaction, 31.2% moderate satisfaction, and 31.3% low or very low satisfaction. For addiction treatment, 31 participants (1.8%) received services, with 37.5% reporting high or very high satisfaction, 28% moderate satisfaction, and 24.5% low or very low satisfaction.

 Lastly, among the 310 participants (18.1%) who received obesity treatment services, 35.7% reported high or very high satisfaction, 30.4% moderate satisfaction, and 33.9% low or very low satisfaction.

Table 3 shows that the highest frequency of reasons for receiving traditional/Islamic medicine services was related to the effectiveness of traditional medicine (51.6%), fear of side effects and ineffectiveness of modern medicine (35.7%). The lowest frequency pertained to lack of or inadequate health insurance coverage (9.25%) and fear of hospitals and physicians (8.12%).

**Table 3:** Reasons to Refer to a Non-specialist to Receive Services

**Type of Service**	**Number (Percentage of Service Recipients)**	**Reason **
Traditional-Islamic medicine	37 (16.3)	General preference
	81 (35.7)	Fear of side effects and medical incapacity of new medicine
	42 (18.5)	Long waiting time for physicians' services
	21 (9.25)	Not having/lack of health insurance coverage
	63 (27.7)	Lack of access or high cost of needed medicine
	117 (51.6)	High effectiveness of traditional treatments
	45 (19.8)	Lack of effective medicine to control the disease
	66 (29.1)	Absence of physicians or specialized medical services
	29 (12.8)	Fear of hospitals and physicians
	75 (33.1)	Recommendation of others
Bonesetter	10 (15.6)	General preference
	15 (23.4)	Long waiting time
	9 (14.1)	Lack of service insurance coverage
	21 (32.8)	Lack of a suitable physician or specialist
	8 (12.5)	Fear of physicians and hospitals
	21 (32.8)	Recommendation of others
Experimental dentistry	37 (30.6)	lower cost
	17 (14.1)	Not having/lack of health insurance coverage
	10 (8.3)	Recommendation of others
	10 (8.3)	High experience of a non-medical person
Abortion	3 (18.7)	Recommendation of others
	13 (81.3)	Legal barriers
Addiction Treatment	10 (40)	Fear of complications and disability caused by common drug treatment
	2 (8)	Long waiting time
	5 (20)	Lack of insurance coverage
	5 (20)	Lack of access/high cost of medicine
	15 (60)	The effectiveness of the medicine provided by a non-physician
	6 (24)	Absence of physician or appropriate specialized services in the place of residence
	5 (20)	Recommendation of others
Obesity Treatment	15 (13.4)	General preference
	9 (8.1)	Long waiting time to receive the services of physicians
	15 (13.4)	Lack of coverage of insurance services
	19 (17)	Absence of physicians or appropriate specialized services
	40 (35.8)	Recommendation of others
	62 (55.4)	Low cost of treatment by non-physician

 Among those who received services from non-medical practitioners, the highest frequency of reasons for seeking services was related to the absence of a suitable physician or specialist (32.8%) and recommendations from others (32.8%). The lowest frequency pertained to fear of physicians and hospitals (12.5%) and lack of health insurance coverage (14.1%).

 Among the 121 individuals who received experimental dental services from non-medical practitioners, 30% of them cited lower costs as the reason for seeking services, 14% cited lack of health insurance coverage, and 8% cited the high experience of the non-physician.

 Among those who received services for abortion from non-physicians, 13 individuals cited legal barriers as the reason for seeking services (81.3%), and the remaining individuals cited recommendations from others (18.7%).

 In addiction treatment services, the highest frequency was related to the effectiveness of the drug provided by a non-physician (60%) and the fear of complications and disability caused by common drug treatment (40%), and the lowest frequency was related to the long waiting time (8%) and lack of insurance coverage (20%).

 Among those who received services for obesity treatment from non-physicians, most individuals cited lower costs for treatment by non-medical practitioners (55.4%) and recommendations from others (35.8%) as the reason for seeking services. The lowest frequency pertained to long waiting times for receiving services from physicians (8.1%) and public preference (13.4%).

 As shown in Table 4, the relationship between at least one visit to a non-medical practitioner versus a medical practitioner for all services, based on demographic characteristics, was examined. First, the univariate analysis of each of the demographic variables with the intended outcome was analyzed. Variables that had a p-value less than 0.200 were included in the multivariate analysis to determine the desired relationship in an adjusted manner compared to other variables. Other variables were not included in the adjusted model due to their higher *P* values.

**Table 4:** Relationship between Seeking Services from Non-medical Practitioners for All Services and the Demographic Characteristics

**Variable**	**Categories**	**OR adjusted**	***P *** **Value**	**Confidence interval (95%)**	
				**Lower Limit**	**Upper Limit**
Marital status	Single	1	-	1	1
	Married	0.93	0.669	0.69	1.27
Gender	Female	1	-	1	1
	Male	0.34	< 0.001	0.26	0.44
Self-expressed socioeconomic status	Bad	1	-	1	1
	Medium	2.02	0.010	1.20	4.01
	Good	2.1	0.035	1.05	3.91
Self-rated health status	very bad	1	-	1	1
	Medium	0.77	0.003	0.59	0.98
	Good	0.63	0.004	0.49	0.86
Job	Unemployed	1	-	1	1
	Government employee	1.03	0.924	0.49	2.20
	Private employee	2.299	0.061	0.99	5.31
Daily activity hours on social networks	Less than an hour	1	-	1	1
	1-2 hours	1.44	0.241	0.92	2.24
	2-3 hours	0.79	0.372	0.54	1.17
	3-4 hours	1.21	0.106	0.79	1.88
	More than 4 hours	2.64	0.020	1.04	3.93

 The baseline for all services was considered to be a visit to a medical practitioner. Based on the findings, the likelihood of visiting a non-medical practitioner for at least one service was significantly lower for men compared to women (OR = 0.34, 95% CI = 0.26 to 0.44).

 Individuals with a good socioeconomic status (OR = 2.1, 95% CI = 1.05 to 3.91) and those with a medium socioeconomic status (OR = 2.02, 95% CI = 1.20 to 4.01) had a higher likelihood of receiving services from non-medical practitioners.

 Those with moderate (OR = 0.77, 95% CI = 0.59 to 0.98) and good health status (OR = 0.63, 95% CI = 0.49 to 0.86) had a lower likelihood of visiting non-medical practitioners. Additionally, individuals who spent more than 4 hours a day on social networks had a 2.64-time higher likelihood of visiting non-medical practitioners compared to those who spent less than 1 hour on social networks (95% CI = 1.04 to 3.93). No significant relationship was observed in other variables (*P* < 0.050)

## Discussion

 This study aimed to investigate the status of receiving health services from non-physicians in Iran and the factors associated with these patient preferences. The results show that many people turn to non-physicians to receive services such as traditional medicine and dental treatments. One of the main reasons for these choices is the belief in the effectiveness of traditional treatments and concern about the side effects of modern medicine. Also, the level of satisfaction with the services of non-physicians varies and the need for more regulation and training is felt to ensure the quality and safety of these services.

 In general, this study demonstrated that the rate of visits to non-physician individuals varies across different services. Although no study has been undertaken to compare the frequency of referrals to non-physicians in different services in general, in health services, referrals to non-physicians may be done due to the need for more special services that physicians cannot provide. For example, in some cases, people may seek non-pharmacological methods to treat their illness. Also, in some health services such as traditional medicine, it is possible that non-physicians have wider information in the provision of services, and thus, a referral to a non-physician is made.

 Regarding visiting non-physicians for traditional medicine services, this study showed that about a quarter of individuals used traditional medicine services in the past year, with only 44% of these services provided by physicians. The highest frequency reason was the effectiveness of traditional medicine drugs, with most individuals receiving services from non-physicians mostly at herbal shops. About one-third of the individuals were highly or very highly satisfied with the services. Traditional medicine has been used for centuries to treat various diseases, but visiting non-physicians for traditional medicine services can have serious health consequences.^10^ Non-physicians may lack the training and expertise necessary to diagnose and treat medical conditions effectively. This can lead to misdiagnosis, delayed treatment, and even worsening conditions.^11^

 Consistent with the present study, a study in the United States showed that 59% of patients needing drugs received their medications and services from non-physicians, with the highest reason being easier access and a positive view of the effectiveness of traditional medicine services provided by non-physicians, which is similar to the findings of this study.^11^ Another study in Iran in 2019 on infertile women showed that about 50% of the individuals had used at least one method of traditional medicine provided by non-physicians in the past year, with more than 50% believing that traditional medicine drugs provided by non-physicians were more effective.^12^

 It seems that, as shown in the study by Ghorbani Nia et al, the availability of services, a positive view of the effectiveness of traditional medicine drugs provided by non-physicians, and fear and skepticism towards modern drugs provided by physicians are reasons for visiting non-physicians,^10^ which was also shown in the present study.

 However, it should be noted that traditional medicine methods may include the use of herbs and other natural substances that can have side effects and interact with other drugs. Non-physicians may not be aware of these interactions and prescribe treatments that may be harmful to the patient. Therefore, providing more tailored traditional medicine services by physicians can ensure the prevention of potential side effects and offer safer services to the target population.

 In the section on orthopedic services by non-physicians, all study participants who received these services in the past year stated that the services were provided by non-physicians. The most frequent reasons for visiting non-physicians were the absence of a specialized physician and recommendations from others. Orthopedic services and bone-setting are among the most common services performed by non-physicians in Iran. Non-physicians performing orthopedic procedures may lack the necessary training and expertise to effectively diagnose and treat complex musculoskeletal conditions, which can lead to misdiagnosis, treatment delays, and even worsening of the condition. Additionally, non-physicians may not be aware of the latest advances and medical techniques in orthopedics, limiting their ability to provide the best possible care to the patients. However, consistent with the findings of this study, a study in Thailand showed that traditional bone-setting by non-physicians is common in many areas of the country due to lack of specialized physicians. In addition to the absence of specialists, recommendations from others to rely on the experience of traditional bone setters were also reasons for people to visit non-physicians for these services.^13^ Another study in Japan showed that most demographic groups used non-physicians for bone-setting services due to the popularity of some non-physician technicians and lack of accessible specialists. Most people were also satisfied with the services they received from non-physicians.^14^ Since non-physician bone-setting methods can lead to serious side effects or exacerbate injuries, greater access to specialized physicians and increased public trust in receiving orthopedic and bone-setting services from physicians can play a crucial role. However, some technical works can be done by non-specialists with a short training.

 Regarding receiving experimental dentistry services, 121 of the total participants in the study stated that they had received these services from non-physicians in the past year, and lower cost and lack of insurance coverage were the most frequent reasons for visiting non-physicians for these services. About half of the people were also satisfied with the services provided by experimental dentists. So far, no study has been conducted to investigate the frequency and reasons for referring to experimental dentists, but it seems that according to the study of various cases, the use of experimental dentistry services may be associated with risks. People who work experimentally and without formal qualifications in this field may not be able to correctly identify what type of treatment is suitable for the patient, and as a result, may cause dental problems to worsen. Also, the use of inappropriate materials and the low quality of experimental dentistry services can lead to serious side effects.

 Regarding obesity treatment services by non-physicians, this study showed that in the past year, about 40% of the individuals received services from non-physicians and non-nutrition experts, with the most frequent reasons being lower treatment costs and recommendations from others. The most frequent service locations were homes and herbal shops. Only 35% of the service recipients were highly or very highly satisfied with the services. Using non-physicians and non-nutrition experts for obesity treatment can pose serious health risks. Obesity treatment requires specialized approaches and attention to the individual’s condition, which can only be effectively managed by specialized physicians and nutrition experts. Using non-invasive methods or medications without physician supervision can lead to serious side effects and, in some cases, threaten the individual’s life. Therefore, individuals should consult specialized physicians for obesity treatment to choose safe and effective methods based on their condition. Consistent with the findings of this study, a structured review study showed that non-physician obesity treatment services had a 70% share among service recipients, with more significant weight loss in physician-led interventions compared to non-physicians.^15^ Another study in the USA showed that lack of insurance support resulted in physicians lacking motivation to provide obesity treatment services, leading people to seek services from non-physicians due to better accessibility and lower costs.^16^ Another study in 11 countries showed that if access to specialized physicians and nutrition experts is provided, the weight loss process will follow an evidence-based path, resulting in more significant weight loss. This important aspect depends on public trust and the widespread acceptance of nutrition experts and medical specialists.^17^ It seems that lower costs and better access to medical and nutritional specialists can significantly reduce people’s visits to non-physicians for obesity treatment. Improved access to medical and nutritional counseling leads to greater public awareness and better health choices. Easier access to medical and nutritional specialists allows individuals to benefit from more specialized and effective methods for weight loss and obesity management,^18^ reducing the need for unqualified and risky weight loss methods, thereby improving health and reducing obesity-related risks.

 Regarding the relationship between visiting non-physicians for traditional medicine services and the demographic factors of participants, this study showed that individuals with self-reported good and very good health statuses were less likely to visit non-physicians compared to those with self-reported poor health statuses. Additionally, individuals with moderate socioeconomic status had a lower likelihood of visiting non-physicians for traditional medicine services compared to those with poor socioeconomic status. Furthermore, individuals who spent 3-4 hours and more than 4 hours daily on social media had a higher likelihood of visiting non-physicians compared to those who spent less than 1 hour on social media.

 A study by Palmer Kelly and colleagues demonstrated that individuals with poorer health and economic status are more compelled to seek traditional cancer treatment services from non-physicians, which aligns with the findings of this study.^19^ Similarly, in another study, Berdahl and colleagues showed that individuals with poorer socioeconomic status and those who experience poor health due to illness are more likely to visit non-physicians due to excessive searching for necessary treatments on social media, which also supports the findings of this study.^20^

 Regarding the relationship between visiting non-physicians for weight loss diet services and the demographic factors of participants, this study showed that married individuals were more likely to visit non-physicians compared to single individuals. Additionally, individuals with self-reported moderate and good socioeconomic statuses had a higher likelihood of visiting non-physicians compared to those with poor socioeconomic status. Furthermore, individuals with self-reported moderate and good health statuses were more likely to visit non-physicians compared to those with poor health status.

 A study by Noriea and colleagues indicated that individuals with poorer self-reported health status and consequently poorer socioeconomic status are more inclined to seek weight loss treatment services from non-physicians because non-physician weight loss services are more expensive, which is consistent with the findings of this study.^21^ Another study in Bangladesh also showed that poor socioeconomic status and the health consequences resulting from diabetes and obesity are barriers to receiving medical services from physicians, leading patients to prefer non-physicians for these services due to easier access and lower costs.

 These insights highlight the significant impact of socioeconomic and health factors on the choice of healthcare providers, especially for traditional medicine and weight loss services. Efforts to improve access to qualified healthcare professionals and address economic barriers could reduce reliance on non-physician services and enhance overall health outcomes.^22^

 This study had some limitations. The sampling method was convenience sampling through online questionnaires. Therefore, generalizing of the findings should be done with caution. The present study was cross-sectional, and the causal path between demographic factors and outcomes was not clear. In this study, the term “bone-setting” was used for orthopedic services, which may have led participants to believe that the service was provided by trained non-physicians. Thus, the generalization of the findings should be done with caution. This study was the first to address the issue of visiting non-physicians in the general community with this sample size. Several services were evaluated together in the questioning process, adding to the comprehensiveness of the study. In the questionnaire, we used the term “ bone setter “ which may create this bias about orthopedic services and make them think that the purpose is only the classification itself and not all orthopedic services.

## Conclusion

 This study demonstrated that some individuals use services provided by non-physicians for various types of services. The reasons for this choice include accessibility, affordability, recommendations from others, and a positive view of the effectiveness of treatments provided by non-physicians. The study showed that factors such as socio-economic status, self-rated health, and hours of activity on social media are among the most important factors associated with the decision to visit non-physicians. Although the use of alternative and herbal medicine is increasingly expanding in different countries, this approach must be based on scientific evidence. Our results showed that use of non-physician services might be considerable. Non-professional practitioners often possess strong communication skills and emotional intelligence, significantly enhancing their ability to persuade others to follow their recommendations, even without scientific backing. Social media amplifies their influence, possibly explaining the increasing attention to their suggestions in recent years. However necessary interventions should be designed to standardize treatments and deal with substandard providers who may be harmful to the health of the community.
